# Joint Exposure to Ambient Air Pollutants Might Elevate the Risk of Small for Gestational Age (SGA) Infants in Wuhan: Evidence From a Cross-Sectional Study

**DOI:** 10.3389/ijph.2022.1605391

**Published:** 2023-01-05

**Authors:** Faxue Zhang, Xupeng Zhang, Yuanyuan Zhong, Shijie Zhu, Gaichan Zhao, Xiaowei Zhang, Tianzhou Li, Yan Zhang, Wei Zhu

**Affiliations:** ^1^ Department of Occupational and Environmental Health, School of Public Health, Wuhan University, Wuhan, China; ^2^ Department of Public Health, School of Public Health, Wuhan University, Wuhan, China; ^3^ Department of Obstetrics and Gynecology, Wuhan Children’s Hospital (Wuhan Maternal and Child Healthcare Hospital), Tongji Medical College, Huazhong University of Science and Technology, Wuhan, China

**Keywords:** cross-sectional study, Wuhan, air pollution score, small for gestational age infant, joint association

## Abstract

**Objective:** To investigate the effect of exposure to multiple ambient air pollutants during pregnancy on the risk of children being born small for gestational age (SGA).

**Methods:** An Air Pollution Score (APS) was constructed to assess the effects of being exposed to six air pollutants simultaneously, PM_2.5_, PM_10_, SO_2_, NO_2_, CO, and O_3_ (referred to as joint exposure). A logistic regression model was applied to estimate the associations of APS and SGA.

**Results:** The adjusted odds ratios (ORs) of SGA per 10 ug/m^3^ increased in APS during the first and second trimesters and the entire pregnancy were 1.003 [95% confidence intervals (CIs): 1.000, 1.007], 1.018 (1.012, 1.025), and 1.020 (1.009, 1.031), respectively. The ORs of SGA for each 10 μg/m^3^ elevated in APS during the whole pregnancy were 1.025 (1.005, 1.046) for mothers aged over 35 years old vs. 1.018 (1.005, 1.031) for mothers aged under 35 years old. Women who were pregnant for the first time were more vulnerable to joint ambient air pollution.

**Conclusion:** In summary, the results of the present study suggested that joint exposure to ambient air pollutants was associated with the increment in the risks of SGA.

## Introduction

Small for gestational age (SGA) is an indicator of fetal development. It applies when infants are born with a birth weight below the 10th percentile for the average weight of newborns of the same gestational age [1]. The prevalence of SGA in developed regions of Europe and America was approximately 10% in 2017 [2]. A previous study estimated that in 2010 more than 32 million SGA infants in low- and middle-income countries and the number of SGA births in China was 1,072,100 (uncertainty intervals: 648,300-1,817,600), meaning China ranked fifth among the top ten countries with the highest numbers of SGA infants [3]. Lower birth weight is one of the major risk factors for neonatal death [4, 5]. In addition, SGA is associated with cognitive impairment in childhood and the development of obesity, type 2 diabetes mellitus, and cardiovascular diseases in adulthood [6, 7].

Air pollution has been an alarming public health problem worldwide in recent years [8]. Emerging studies have shown that short- and long-term exposure to air pollution is associated with elevated mortality and morbidity of various diseases [9, 10]. The relationship between air pollution and SGA had been extensively assessed in recent years [11, 12]. A study conducted in Huangshi, China found that fine particulate matter (PM_2.5_) and inhalable particles (PM_10_) were positively linked with increased risks of SGA during the entire pregnancy [13]. In the Eunice Kennedy Shriver National Institute of Child Health and Human Development (NICHD) Consecutive Pregnancy Study, the significant effects of PM_2.5_, PM_10_, nitrogen dioxide (NO_2_), sulfur dioxide (SO_2_), and carbon monoxide (CO) on SGA were found in the third trimester [14]. However, the overwhelming majority of research to date has applied single-pollutant models, which often ignore the fact that multiple people are exposed to ambient air pollution simultaneously. The combined health effect of particulate matter and gaseous pollutants might be widely divergent from that of individual air pollutants [15, 16]. Therefore, we propose a novel indicator [17], the Air Pollution Score (APS), to aid in comprehensively considering the effects of PM_2.5_, PM_10_, NO_2_, SO_2_, CO, and ozone (O_3_), in evaluating the associations of mixed exposure to air pollution and SGA.

This study applied logistic regression models to estimate the associations between joint exposure to multiple ambient air pollutants and SGA in Wuhan, China. We also performed subgroup analyses to explore potentially susceptible populations and the times of year when people are most vulnerable.

## Methods

### Study Design and Population

**FIGURE 1:**
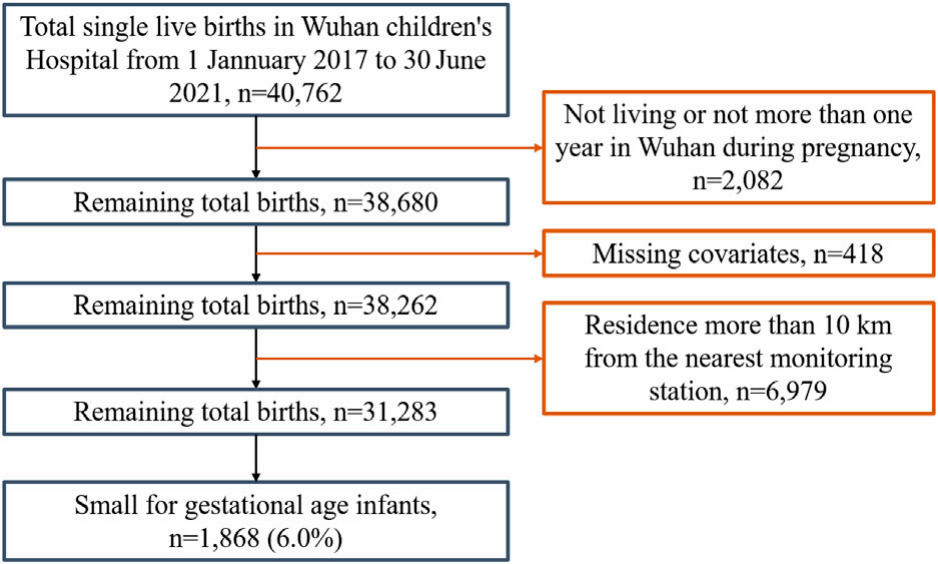
Flow chart of the study population selection (Wuhan, China, 2022).

**FIGURE 2:**
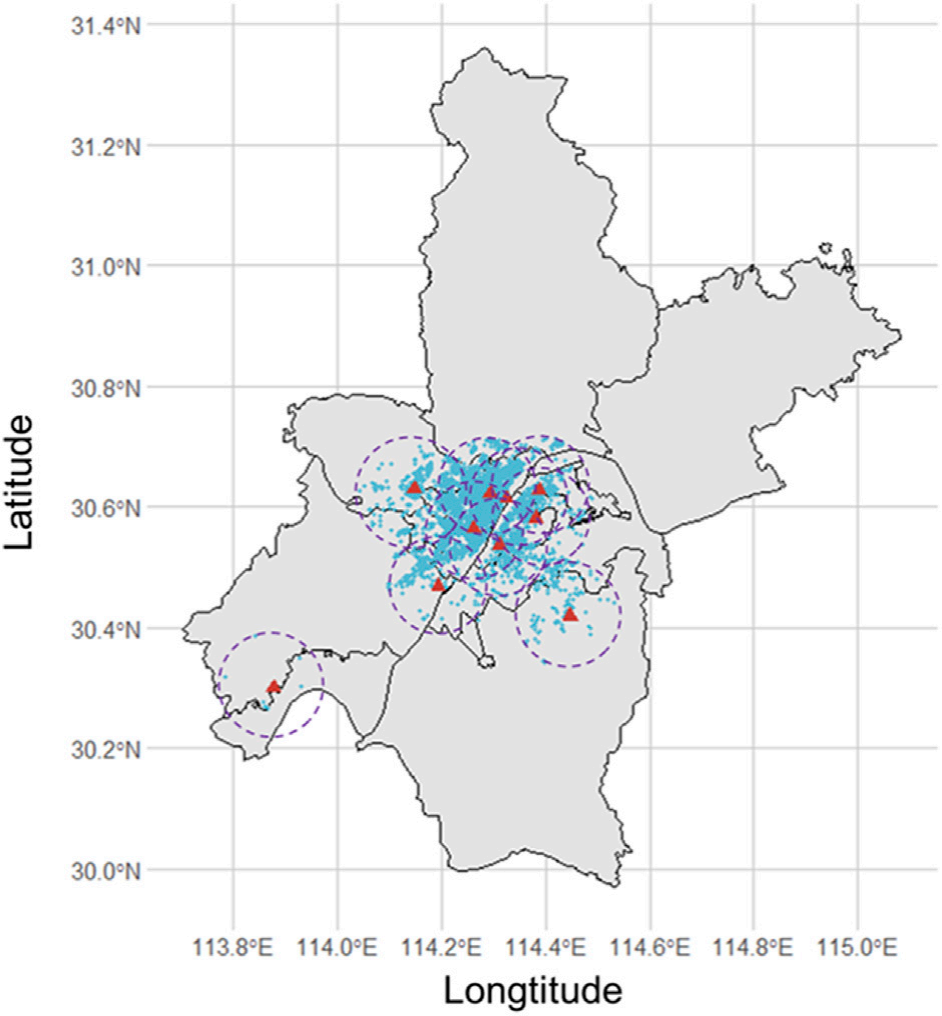
The distribution of pregnant women whose home addresses are less than 10 km from the nearest monitoring station (Wuhan, China, 2022).

Wuhan Children’s Hospital is a large-scale specialized hospital located in the Jiang’an district of Wuhan, China. This area is one of the central urban areas of Wuhan city, serving pregnant women and the delivery needs of the whole city (Supplementary Figure S1). Data on mothers and live newborns were collected from Wuhan Children’s Hospital from 1 January 2017, to 30 June 2021. After screening by inclusion and exclusion criteria, a total of 31,283 gravidas and their offspring were involved in the study (Figure 1). Their geographical distributions are shown in Figure 2. We obtained variables of interest from the hospital’s delivery register, including each gravida’s residential address and duration, work status, educational attainment, maternal age, the number of pregnancies and parity, high-risk factors during pregnancy (for example, placental abruption, placenta previa, gestational hypertension, preeclampsia, eclampsia, oligohydramnios, and gestational diabetes, etc.), gestational age, date of delivery, birth weight and sex of newborns. Furthermore, we also deduced gravidas’ conception seasons [warm (April to September) and cold (October to March of the next year)] based on the date of delivery and gestational age.

### Assessment of SGA

The weight of the newborns was measured within 1 hour after birth. SGA was defined as infants with a birth weight lower than the 10th percentile of the average weight at the same gestational age [18]. Compared with the traditional indicator (low birth weight, LBW), SGA can more accurately reflect the development of the infant by considering development time in the womb. According to growth standard curves of the birth weights of Chinese newborns of different gestation [19], the incidence rate of SGA was approximately 6.0% in this study.

### Definition of the Air Pollution Score

The daily mean concentrations of PM_2.5_, PM_10_, SO_2_, NO_2_, CO, and O_3_ were collected from 10 national environmental monitoring stations based in the Wuhan Municipal Bureau of Ecological Environment (http://hbj.wuhan.gov.cn/hjsj/). The concentration of air pollution published by the environmental monitoring station closest to the home address of gravida was used as the individual exposure level (Figure 1).

APS is a novel indicator for assessing the joint effects of being exposed to multiple ambient air pollutants simultaneously and the health outcomes that result from it [17]. In the current study, the air pollution score was calculated by considering concentrations of six air pollutants, weighted by multivariable-adjusted risk estimates (β coefficients) on SGA. The formula was as follows:

$$APS=\frac{6\times \left(\beta_{{PM}_{2.5}}\times {PM}_{2.5}+\beta_{{PM}_{10}}\times {PM}_{10}+\beta_{{SO}_{2}}\times {SO}_{2}+\beta_{{NO}_{2}}\times {NO}_{2}+\beta_{CO}\times CO+\beta_{O_{3}}\times O_{3}\right)}{\beta_{{PM}_{2.5}}+\beta_{{PM}_{10}}+\beta_{{SO}_{2}}+\beta_{{NO}_{2}}+\beta_{CO}+\beta_{O_{3}}}$$

Where $\beta_{{PM}_{2.5}},\beta_{{PM}_{10}},\beta_{{SO}_{2}},\beta_{{NO}_{2}},\beta_{CO}$ and $\beta_{O_{3}}$ were the regression coefficients calculated from each single-pollutant model ${PM}_{2.5},{PM}_{10},{SO}_{2},{NO}_{2},\;CO$ and $O_{3}$ were the concentration of each air pollutant. The higher APS of the pregnant woman indicated that they had been exposed to higher joint ambient air pollutants.

### Statistical Analysis

According to previous epidemiological studies [20], a pregnancy period is divided into the first trimester (1–12 weeks), second trimester (13–26 weeks), and third trimester (27 weeks to birth). A two-stage analysis strategy was developed to investigate the associations between joint ambient air pollution and SGA. In the first stage, the links between six air pollutants and SGA were assessed by performing multiple logistic regression in separate models, and then APS was calculated. At the second stage, the effects of APS on SGA were evaluated to reflect the hazards of mixed air pollution exposure. Several potential confounders were selected and adjusted in these models, including age [year old (<35, 35+)], pregnancy (=1, >1), parity (=1, >1), educational attainment [year (≤9, 10–12, ≥13)], work status (yes, no), high-risk factor during pregnancy (yes, no) and neonatal sex (boy, girl). Moreover, we separated APS into five quintiles (Q1, Q2, Q3, Q4, and Q5) and estimate the ORs compared with the first quintiles (Q1) to evaluate the potential linear trend between APS and SGA. Subgroup analyses were stratified by age, pregnancy, parity, and conception season to detect vulnerable populations and periods. To assess the robustness of the association between APS and SGA, we conducted several sensitivity analyses: 1) re-calculating APS removing one air pollutant at a time; 2) assessment of SGA using internal standard; 3) co-adjusting for two trimesters in the same model; 4) co-controlling for two air pollutants in the same model.

Consistent with previous studies, the effects of air pollution and APS on SGA were reported odds ratio (ORs) with 95% confidence intervals (CIs). All statistical analyses were performed using R software (version 4.0.3), All *P*-values for the tests were two-sided and *P*-values < 0.05 were considered statistically significant.

## Results

Table 1 shows the characteristics of mothers and newborns in Wuhan, China. In both the case group and control group, lying-in women who were aged under 35 years old, had primiparity, higher educational level, were working, and had high-risk factors, and those who gave birth to boys accounted for more than half of the total participants. However, a higher proportion of first-time pregnant mothers were observed in the SGA group compared with the normal group (57.3% vs. 45.9%). Table 2 shows the adjusted odds ratios (ORs) and 95% CIs for SGA associated with a 10 ug/m^3^ increase in air pollutants during different exposure periods. Exposure to SO_2_ appeared the strongest effects on SGA in the second trimester [OR = 1.320, 95% CIs (1.110, 1.570)].

**TABLE 1:** The characteristics of mothers and newborns (Wuhan, China, 2022).

	Control (*n* = 29,415)	SGA (*n* = 1868)
Age (years), n (%)		
<35	18,522 (63.0%)	1,321 (70.7%)
35+	10,893 (37.0%)	547 (29.3%)
Pregnancy, n (%)		
=1	13,515 (45.9%)	1,071 (57.3%)
>1	15,900 (54.1%)	797 (42.7%)
Parity, n (%)		
=1	18,914 (64.3%)	1,465 (78.4%)
>1	10,501 (35.7%)	403 (21.6%)
Educational attainment (years), n (%)		
≤9	5,363 (18.2%)	324 (17.3%)
10–12	2,164 (7.4%)	133 (7.1%)
≥13	21,888 (74.4%)	1,411 (75.5%)
Work status, n (%)		
Yes	17,307 (58.8%)	1,098 (58.8%)
No	12,108 (41.2%)	770 (41.2%)
High-risk factor, n (%)		
Yes	21,652 (73.6%)	1,334 (71.4%)
No	7,763 (26.4%)	534 (28.6%)
Neonatal sex, n (%)		
Boy	15,595 (53.0%)	1,062 (56.9%)
Girl	13,820 (47.0%)	806 (43.1%)

**TABLE 2:** The odds ratios and 95% confidence intervals for small for gestational age infants associated with a 10 μg per cubic meter increase in air pollutants (Wuhan, China, 2022).

	Trimester 1	Trimester 2	Trimester 3	Entire pregnancy
PM_2.5_	1.009 (0.982, 1.036)	1.077 (1.049, 1.106)	0.988 (0.962, 1.014)	1.116 (1.053, 1.181)
PM_10_	1.009 (0.986, 1.033)	1.062 (1.037, 1.087)	1.010 (0.987, 1.033)	1.069 (1.030, 1.109)
SO_2_	1.045 (0.882, 1.237)	1.320 (1.110, 1.570)	0.977 (0.815, 1.172)	1.261 (0.969, 1.640)
NO_2_	1.034 (0.989, 1.080)	1.087 (1.040, 1.136)	0.970 (0.929, 1.013)	1.085 (1.008, 1.169)
CO	1.003 (1.000, 1.006)	1.005 (1.002, 1.008)	1.000 (0.997, 1.002)	1.001 (1.000, 1.002)
O_3_	0.977 (0.953, 1.001)	0.943 (0.921, 0.967)	1.035 (1.010, 1.060)	0.918 (0.866, 0.973)

Table 3 exhibits adjusted odds ratios (ORs) and 95% confidence intervals for the associations of SGA and APS. A 10 ug/m^3^ increase in APS was positively associated with elevated risk of SGA, with corresponding ORs of 1.018 (95% CIs: 1.012, 1.025) and 1.020 (95% CIs: 1.009, 1.031) in the second trimester and the entire pregnancy, respectively. Compared with the first quintile of APS in different pregnancy periods. There was a significant relationship between APS and SGA detected in the second trimester [Q5 vs. Q1, OR = 1.427 (95% CIs: 1.231, 1.653)] and entire pregnancy [Q5 vs. Q1, OR = 1.346 (95% CIs: 1.162, 1.558)]. In general, the higher the concentrations of APS, the larger their effect estimates on SGA (*p* for trend <0.05).

**TABLE 3:** The adjusted odd ratios and 95% confidence intervals for air pollution scores with the risk of small for gestational age infants in Wuhan (Wuhan, China, 2022).

	Air pollution score (quintiles)					ORs for per 10 ug/m^3^ increase	*p* for trend
	Q1	Q2	Q3	Q4	Q5		
Trimester 1	1	1.042 (0.893, 1.216)	1.247 (1.075, 1.447)	1.206 (1.038, 1.401)	1.105 (0.949, 1.288)	1.003 (1.000, 1.007)	0.046
Trimester 2	1	1.054 (0.902, 1.232)	1.118 (0.959, 1.304)	1.133 (0.972, 1.320)	1.427 (1.231, 1.653)	1.018 (1.012, 1.025)	<0.001
Trimester 3	1	1.157 (1.001, 1.336)	1.016 (0.876, 1.179)	1.006 (0.868, 1.168)	0.862 (0.738, 1.005)	0.998 (0.996, 0.999)	0.013
Entire pregnancy	1	1.129 (0.972, 1.311)	0.987 (0.845, 1.152)	0.993 (0.850, 1.160)	1.346 (1.162, 1.558)	1.020 (1.009, 1.031)	0.003

Note: Q, quintile.

Table 4 compares the adjusted ORs per 10 ug/m^3^ increment of APS in different subgroups during the four exposure windows. Pregnant women with advanced maternal age seemed to be more susceptible to exposure to mixed air pollutants than younger women. The ORs of SGA per 10 ug/m^3^ elevated in APS during the second trimester and entire pregnancy were 1.022 (95% CIs: 1.010, 1.033) for mothers aged over 35 years old and 1.016 (95% CIs: 1.008, 1.024) for mothers aged under 35 years old, and 1.025 (95% CIs: 1.005, 1.046) vs. 1.018 (95% CIs: 1.005, 1.031), respectively. Results showed that women experiencing their first pregnancy were more vulnerable to joint ambient air pollution than women who have had multiple pregnancies. For the conception season, the increment of APS related to an increase in the risk of SGA corresponded with the warm season only. 

**TABLE 4:** The adjusted odd ratios and 95% confidence intervals for a 10 μg per cubic meter increase in air pollution scores with the risk of small for gestational age infants in Wuhan (Wuhan, China, 2022).

	Trimester 1	Trimester 2	Trimester 3	Entire pregnancy
Age (years)				
<35	1.004 (1.000, 1.009)	1.016 (1.008, 1.024)	0.998 (0.996, 1.000)	1.018 (1.005, 1.031)
35+	1.002 (0.995, 1.009)	1.022 (1.010, 1.033)	0.998 (0.995, 1.001)	1.025 (1.005, 1.046)
Pregnancy				
=1	1.005 (1.000, 1.010)	1.020 (1.011, 1.028)	0.997 (0.995, 0.999)	1.025 (1.010, 1.040)
>1	1.001 (0.995, 1.007)	1.016 (1.006, 1.027)	0.999 (0.996, 1.001)	1.014 (0.998, 1.031)
Parity				
=1	1.005 (1.000, 1.009)	1.018 (1.010, 1.025)	0.997 (0.995, 0.999)	1.019 (1.006, 1.031)
>1	0.999 (0.991, 1.007)	1.019 (1.005, 1.034)	1.001 (0.998, 1.005)	1.026 (1.003, 1.050)
Season				
Warm	1.018 (1.011, 1.025)	1.026 (1.017, 1.036)	0.993 (0.991, 0.996)	1.029 (1.013, 1.046)
Cold	1.006 (0.998, 1.013)	1.004 (0.992, 1.016)	0.998 (0.995, 1.002)	1.007 (0.991, 1.023)

After re-calculating APS by removing one air pollutant at a time, the links between APS and SGA were still statistically significant in the second trimester and the entire pregnancy (Supplementary Table S1). We re-estimated the associations between SGA (internal standard, as seen in Supplementary Table S2) and air pollutants. Compared with previous results, the associations still exist, although the impact was slightly smaller (Supplementary Table S3). In two-trimester and two-pollutant models, the relationship between air pollution and SGA did not change significantly (Supplementary Tables S4, S5). The several sensitivity analyses showed our results were robust.

## Discussion

In this study, we used APS to estimate the effect of joint exposure to ambient air pollutants on SGA by unconditional logistic regression models. We found significant positive associations between APS-SGA in the first, second, and third trimesters, as well as for the entire pregnancy. Compared with the first quintile of APS, the effect estimates were strongest in the fifth quintile. Gravidas, being aged over 35 years old, multiparas, women experiencing their first pregnancy, and conception taking place in the warm season were shown to make pregnancies more vulnerable to mixed air pollution during the second trimester and whole pregnancy. These findings contribute to the existing body of knowledge on the relationship between mixed air pollution and SGA and provide valuable epidemiological evidence for policymakers and will help better maternal and child health.

A number of studies have revealed that exposure to air pollution during pregnancy increases the risk of SGA [21, 22]. For example, a 1 ug/m^3^ increase in PM_2.5_ during the second trimester corresponded with a 1.9% (95% CIs: 0.9%, 2.8%) increased incidence of SGA [13]. The third quartile of PM_10_ in the whole pregnancy compared with the first quartile was linked with SGA [OR = 1.38 (95% CIs: 1.00, 1.90)] [23]. The adjusted ORs for SGA associated with per interquartile range (IQR) increased in SO_2_, NO_2,_ and O_3_ during the entire pregnancy were 1.02 (95% CIs: 1.01, 1.03), 1.08 (95% CIs: 1.04, 1.12), and 1.14 (95% CIs: 1.11, 1.17), respectively [12]. A 1 part per million (ppm) increase in the concentration of CO in the first month of pregnancy was significantly related to SGA [OR = 1.06 (95% CIs: 1.01, 1.10)] [24]. However, in the current paper, we found negative correlations of O_3_. Possible explanations is the different ethnicities of patients and the fact that the combination of air pollution in specific regions differed. Animal experiments using an ozone exposure chamber are warranted to investigate the associations between O_3_ and SGA.

In recent years, more research attention has been paid to the health effects of mixed pollutant exposure due to the high correlation between them and the fact that they might be emitted simultaneously from the same sources [25, 26]. However, few studies have estimated the joint association between ambient air pollutants and SGA. We comprehensively constructed a novel indicator, APS, reflecting the combined effects of PM_2.5_, PM_10_, SO_2_, NO_2_, CO, and O_3_, to evaluate the association between joint exposure to air pollutants and SGA. Although NO_2_, CO, and O_3_ appeared non-positive effects on SGA in the single-pollutant models, we found that APS was significantly associated with an elevated risk of SGA in each exposure window. Therefore, APS might have the capacity to provide more comprehensive measures of health assessment than individual air pollutants. Prior studies had utilized similar methods by accounting for a combination of common coexisting pollutants to assess the combined effects of multi-pollutant exposures on health outcomes [27, 28]. Hong et al. proposed an index calculated by summing each air pollutant concentration divided by its mean and observed that the new index was more strongly associated with all-cause mortality than individual air pollutants [28]. An analogous algorithm, environmental risk score (ERS) was also used to estimate the relationships between joint exposure to environmental pollutants and serum lipid levels in people in the US [29]. A statistical approach to comprehensively summarizing air pollutant concentrations could efficiently evaluate the combined effects of pollutants as they are highly correlated [17].

Previous studies have indicated that prenatal exposure to air pollution is associated with a smaller head circumference, shorter body length, and lower weight in newborns [30–32]. The potential biological mechanism of air pollution on SGA remains unclear. However, there were several theories worth mentioning, including: 1) that air pollution might trigger oxidative stress and inflammatory reaction, reducing nutrition and the exchange of gases in the placenta and inducing endocrine disorders in the maternal body [33]; 2) prenatal exposure to air pollution could increase maternal susceptibility to infections, impairing fetal growth [34]; and 3) exposure to air pollution is capable of reducing the content of mitochondrial DNA (mtDNA), which has been related to lower infant birth weight and more muscular oxidative stress [35, 36]. The potential mechanism of joint exposure to air pollutants and increased risks of SGA is still unknown. We speculated that there might be additive effects in various air pollutants on SGA because of their similar biological function pathways, such as oxidative stress and inflammatory reaction.

We observed that when the maternal age is over 35 years old the pregnancy seems to be more vulnerable to APS in the second trimester and whole pregnancy, which can be explained due to the problem of hypoxia in older pregnant women being more prominent due to the increase in air pollution levels and umbilical artery vasoconstriction [37, 38]. Younger women might generally be more capable of relieving the oxidative stress induced by air pollution [39]. Slightly stronger associations between APS and SGA were found in women who were having their first pregnancy than those who have had multiple pregnancies, during the entire pregnancy, with corresponding ORs of 1.025 (95% CIs: 1.010, 1.040) vs. 1.014 (95% CIs: 0.998, 1.031). Prior studies have also revealed that women with a history of pregnancy and childbirth were associated with an increase in the birth weights of newborns [40, 41]. Prefumo et al. found that permanent changes in maternal blood vessels might persist after a successful pregnancy and that these changes in the physiological structure also changed the hemodynamics of women during the second pregnancy, which was more conducive to the material exchange between mother and fetus [42]. Previous studies have evidenced that the placental efficiency of primipara is relatively low, which affects the development of the surface density of the microcotyledon; whereas with an increase in the number of parities, the surface density elevates accordingly [43–45], which might mitigate the hazards of air pollution to a certain degree. The specific mechanism of how both parity and APS influence neonatal birth weight needs to be further explored. In this paper, we found that when women conceived in the warm season they were more susceptible to APS than in the cold season. Similar results were found in a study conducted in Guangzhou, China [12]. Wang et al. observed that an IQR increase in PM_2.5_, PM_10_, NO_2_, SO_2_, and O_3_ significantly elevated SGA incidence among women whose conception season was summer or fall [12]. However, a study conducted in Canada found there was no seasonal pattern between air pollution and SGA [32], meaning weather conditions might only partly explain the difference. Wuhan and Guangzhou belong to subtropical oceanic monsoon climates, and women conceived in the warm season experience relatively cold weather during pregnancy. A previous study pointed out that people living in Wuhan and Guangzhou were more vulnerable to cold weather than those in the cities of northern China [46]. Therefore, simultaneous exposure to lower temperatures and humidity might strengthen the adverse effect of air pollution on SGA. Besides, the lower duration of sunshine in the cold season has been associated with lower maternal vitamin D levels, and vitamin D deficiency was a risk factor for low birth weight in infants [47].

According to the information we currently have, this is the first study to explore the associations between joint exposure to ambient air pollutants and SGA in China. These findings deepen our understanding of the impact of mixed air pollution and indicate that new prevention strategies to curb various air pollutants together are needed. There are several limitations of this study that must be acknowledged: 1) data on air pollution reported by the environmental monitoring station closest to the home address of gravida was used to measure individual exposure, which could lead to misclassifications to some extent; 2) we regarded air pollutants as continuous variables when constructing APS, however, the link between air pollution and SGA might not be linear; 3) we did not adjust for some potential confounders such as work hazards, smoking, drinking, diet etc., due to data on these not being available; 4) this study was conducted in Wuhan, so caution should be taken when conclusions were extended to other regions; 5) although choosing which hospital to give birth is the decision of the patient, there might be selection bias because the results only relate to one hospital; 6) the effects of O_3_ might be confounded by other air pollutants, so animal experiments using an ozone exposure chamber are warranted to investigate the true associations between O_3_ and SGA.

### Conclusion

In summary, our study suggested that joint exposure to ambient air pollutants including PM_2.5_, PM_10_, SO_2_, NO_2_, CO, and O_3_, evaluated as APS, were associated with an increase in risks of SGA. Women who were of advanced maternal age, primipara, first pregnancy, and conceived in the warm season were more susceptible to APS. Consequently, our results might provide valuable epidemiological evidence for policymakers and public health departments to comprehensively curb air pollution and protect neonatal health.
