# Supplementary material for: Joint Exposure to Ambient Air Pollutants Might Elevate the Risk of Small for Gestational Age (SGA) Infants in Wuhan: Evidence From a Cross-Sectional Study
Source: Int J Public Health. 2023 Jan 5;67:1605391. doi: 10.3389/ijph.2022.1605391 (PMC9849243; doi:10.3389/ijph.2022.1605391)
Supplement: Supplementary file 1 [file DataSheet1.docx]

**Supplementary Material**

Joint exposure to various ambient air pollutants might elevate the risks of small for gestational age infants in Wuhan Evidence from a cross-sectional study

Faxue Zhang ^a, 1^, Xupeng Zhang ^b, 1^, Yuanyuan Zhong ^c^, Shijie Zhu ^a^, Xiaowei Zhang ^a^, Gaichan Zhao ^b^, Tianzhou Li ^a^, Yan Zhang ^c *^, Wei Zhu ^a **^^[[1]](#footnote-1)^

*^a^* *Department of Occupational and Environmental Health, School of Public Health, Wuhan University, Wuhan 430071, China*

*^b^ Department of Public Health, School of Public Health, Wuhan University, Wuhan 430071, China*

*^c^* *Department of Obstetrics and Gynecology, Wuhan Children’s Hospital (Wuhan Maternal and Child Healthcare Hospital), Tongji Medical College, Huazhong University of Science and Technology, Wuhan 430014, China*

Table of contents

**Table S1.** The adjusted odds ratios (ORs) and 95% confidence interval for a 10 ug/m^3^ increased in APS with the risk of incident SGA in sensitivity analyses

**Table S2.** The 10^th^ percentile of infant weight at different gestational weeks in China and Wuhan

**Table S3.** The odds ratios (ORs) and 95% confidence interval for SGA (internal standard) associated with per 10 ug/m^3^ increased in air pollutants

**Table S4.** The adjusted odds ratios (ORs) and 95% confidence interval for per 10 ug/m^3^ increased in APS with the risk of incident SGA in two-trimester model

**Table S5.** The adjusted odds ratios (ORs) and 95% confidence interval for per 10 ug/m^3^ increased in air pollutants with the risk of incident SGA in two-pollutant model

**Fig. S1.** The locations of environmental monitoring stations and residences of gravidas

| Table S1 The adjusted odd ratios (ORs) and 95% confidence interval for a 10 ug/m^3^ increased in APS with the risk of incident SGA | | | | | | |
| --- | --- | --- | --- | --- | --- | --- |
|  | Air pollution scores | | | | | |
|  | Without PM_2.5_ | Without PM_10_ | Without SO_2_ | Without NO_2_ | Without CO | Without O_3_ |
| Trimester 1 | 1.004 (1.000, 1.009) | 1.004 (1.000, 1.009) | 1.002 (1.000, 1.004) | 1.003 (1.000, 1.006) | 1.005 (0.999, 1.011) | 1.007 (0.998, 1.015) |
| Trimester 2 | 1.023 (1.014, 1.031) | 1.023 (1.015, 1.032) | 1.009 (1.006, 1.012) | 1.021 (1.014, 1.029) | 1.024 (1.015, 1.033) | 1.030 (1.019, 1.041) |
| Trimester 3 | 0.998 (0.997, 0.999) | 0.997 (0.995, 1.000) | 1.000 (1.000, 1.000) | 1.001 (1.000, 1.002) | 0.997 (0.995, 0.999) | 0.988 (0.975, 1.000) |
| Entire pregnancy | 1.023 (1.010, 1.037) | 1.026 (1.011, 1.041) | 1.011 (1.005, 1.017) | 1.023 (1.011, 1.036) | 1.024 (1.011, 1.038) | 1.036 (1.016, 1.056) |

| Table S2 The 10th percentile of infant weight at different gestational weeks in China and Wuhan | | | | | |
| --- | --- | --- | --- | --- | --- |
| Gestational Week | Boys (g) | |  | Girls (g) | |
|  | China | Wuhan |  | China | Wuhan |
| 26 | 719 | 365 |  | 654 | 307 |
| 27 | 809 | 507 |  | 745 | 455 |
| 28 | 910 | 668 |  | 844 | 624 |
| 29 | 1023 | 843 |  | 951 | 807 |
| 30 | 1150 | 1030 |  | 1068 | 997 |
| 31 | 1292 | 1231 |  | 1198 | 1192 |
| 32 | 1451 | 1444 |  | 1344 | 1394 |
| 33 | 1628 | 1669 |  | 1509 | 1604 |
| 34 | 1823 | 1908 |  | 1695 | 1825 |
| 35 | 2033 | 2156 |  | 1902 | 2059 |
| 36 | 2258 | 2406 |  | 2125 | 2305 |
| 37 | 2487 | 2643 |  | 2357 | 2543 |
| 38 | 2701 | 2835 |  | 2579 | 2738 |
| 39 | 2874 | 2954 |  | 2762 | 2863 |
| 40 | 3002 | 3045 |  | 2896 | 2951 |
| 41 | 3100 | 3138 |  | 3005 | 3032 |
| 42 | 3188 | 3227 |  | 3101 | 3111 |

| Table S3 The odds ratios (ORs) and 95% confidence interval for SGA (internal standard) associated with per 10 ug/m^3^ increased in air pollutants | | | | |
| --- | --- | --- | --- | --- |
|  | Trimester 1 | Trimester 2 | Trimester 3 | Entire pregnancy |
| PM_2.5_ | 0.999 (0.978, 1.021) | 1.054 (1.032, 1.077) | 0.989 (0.968, 1.010) | 1.064 (1.016, 1.114) |
| PM_10_ | 0.997 (0.978, 1.016) | 1.043 (1.024, 1.063) | 1.001 (0.983, 1.019) | 1.034 (1.004, 1.065) |
| SO_2_ | 1.052 (0.919, 1.205) | 1.256 (1.092, 1.444) | 0.972 (0.841, 1.124) | 1.213 (0.981, 1.500) |
| NO_2_ | 1.008 (0.973, 1.044) | 1.066 (1.029, 1.105) | 0.962 (0.930, 0.996) | 1.031 (0.971, 1.093) |
| CO | 1.001 (0.999, 1.004) | 1.003 (1.001, 1.006) | 1.000 (1.000, 1.001) | 1.001 (1.000, 1.002) |
| O_3_ | 0.988 (0.969, 1.007) | 0.955 (0.937, 0.974) | 1.027 (1.007, 1.047) | 0.944 (0.901, 0.989) |

| Table S4 The adjusted odds ratios (ORs) and 95% confidence interval for per 10 ug/m^3^ increased in APS with the risk of incident SGA in two-trimester model | | | |
| --- | --- | --- | --- |
|  | Trimester 1 | Trimester 2 | Trimester 3 |
| +Trimester 1 | - | 1.018 (1.011, 1.024) | 0.998 (0.995, 1.000) |
| +Trimester 2 | 1.002 (0.998, 1.006) | - | 0.998 (0.996, 1.000) |
| +Trimester 3 | 1.000 (0.995, 1.005) | 1.018 (1.012, 1.025) | - |

| Table S5 The adjusted odds ratios (ORs) and 95% confidence interval for per 10 ug/m^3^ increased in air pollutants with the risk of incident SGA in two-pollutant model | | | | | | |
| --- | --- | --- | --- | --- | --- | --- |
|  | PM_2.5_ | PM_10_ | SO_2_ | NO_2_ | CO | O_3_ |
| Trimester 1 |  |  |  |  |  |  |
| + PM_2.5_ | - | 1.009 (0.965, 1.054) | 1.015 (0.817, 1.260) | 1.052 (0.985, 1.125) | 1.006 (1.001, 1.011) | 0.941 (0.897, 0.986) |
| + PM_10_ | 1.001 (0.952, 1.052) | - | 1.010 (0.820, 1.242) | 1.057 (0.982, 1.139) | 1.004 (1.000, 1.008) | 0.969 (0.937, 1.002) |
| + SO_2_ | 1.007 (0.974, 1.042) | 1.008 (0.980, 1.038) | - | 1.042 (0.986, 1.101) | 1.004 (1.000, 1.007) | 0.974 (0.947, 1.001) |
| + NO_2_ | 0.986 (0.947, 1.026) | 0.985 (0.947, 1.025) | 0.949 (0.767, 1.175) | - | 1.003 (0.999, 1.006) | 0.979 (0.944, 1.015) |
| + CO | 0.964 (0.922, 1.008) | 0.987 (0.956, 1.019) | 0.911 (0.735, 1.129) | 1.010 (0.957, 1.067) | - | 0.989 (0.955, 1.024) |
| + O_3_ | 0.953 (0.906, 1.003) | 0.988 (0.957, 1.021) | 0.957 (0.787, 1.163) | 1.005 (0.941, 1.072) | 1.002 (0.998, 1.006) | - |
| Trimester 2 |  |  |  |  |  |  |
| + PM_2.5_ | - | 1.015 (0.971, 1.061) | 0.976 (0.781, 1.219) | 0.979 (0.916, 1.046) | 1.001 (0.999, 1.003) | 1.001 (0.955, 1.048) |
| + PM_10_ | 1.062 (1.010, 1.116) | - | 1.031 (0.831, 1.278) | 0.977 (0.907, 1.053) | 1.002 (1.000, 1.004) | 0.972 (0.940, 1.005) |
| + SO_2_ | 1.079 (1.045, 1.115) | 1.060 (1.030, 1.090) | - | 1.067 (1.011, 1.126) | 1.003 (1.000, 1.006) | 0.950 (0.925, 0.977) |
| + NO_2_ | 1.087 (1.045, 1.131) | 1.073 (1.031, 1.116) | 1.141 (0.921, 1.413) | - | 1.003 (1.000, 1.005) | 0.947 (0.913, 0.983) |
| + CO | 1.068 (1.038, 1.099) | 1.053 (1.027, 1.080) | 1.209 (0.993, 1.472) | 1.066 (1.015, 1.119) | - | 0.953 (0.928, 0.979) |
| + O_3_ | 1.078 (1.025, 1.133) | 1.042 (1.009, 1.076) | 1.119 (0.918, 1.366) | 1.010 (0.945, 1.080) | 1.002 (1.000, 1.004) | - |
| Trimester 3 |  |  |  |  |  |  |
| + PM_2.5_ | - | 1.076 (1.029, 1.124) | 1.045 (0.833, 1.311) | 0.965 (0.904, 1.031) | 1.000 (0.999, 1.001) | 1.088 (1.041, 1.138) |
| + PM_10_ | 0.918 (0.871, 0.967) | - | 0.895 (0.711, 1.127) | 0.865 (0.801, 0.934) | 0.998 (0.994, 1.002) | 1.081 (1.046, 1.117) |
| + SO_2_ | 0.984 (0.952, 1.017) | 1.018 (0.989, 1.048) | - | 0.960 (0.910, 1.012) | 1.000 (0.998, 1.002) | 1.042 (1.014, 1.071) |
| + NO_2_ | 1.004 (0.964, 1.045) | 1.076 (1.033, 1.121) | 1.082 (0.866, 1.351) | - | 1.000 (0.999, 1.001) | 1.048 (1.011, 1.087) |
| + CO | 0.988 (0.961, 1.017) | 1.021 (0.989, 1.054) | 0.988 (0.814, 1.198) | 0.971 (0.929, 1.015) | - | 1.035 (1.010, 1.061) |
| + O_3_ | 1.068 (1.017, 1.122) | 1.062 (1.029, 1.095) | 1.128 (0.923, 1.379) | 1.032 (0.968, 1.101) | 1.000 (0.999, 1.001) | - |
| Entire Pregnancy |  |  |  |  |  |  |
| + PM_2.5_ | - | 1.023 (0.950, 1.100) | 0.989 (0.727, 1.345) | 0.974 (0.881, 1.078) | 1.001 (0.999, 1.002) | 0.992 (0.910, 1.081) |
| + PM_10_ | 1.083 (0.967, 1.213) | - | 0.994 (0.730, 1.353) | 0.970 (0.872, 1.080) | 1.001 (0.999, 1.002) | 0.966 (0.897, 1.040) |
| + SO_2_ | 1.117 (1.046, 1.192) | 1.069 (1.025, 1.116) | - | 1.069 (0.985, 1.160) | 1.001 (1.000, 1.002) | 0.925 (0.871, 0.983) |
| + NO_2_ | 1.131 (1.046, 1.223) | 1.081 (1.024, 1.140) | 1.141 (0.852, 1.529) | - | 1.001 (1.000, 1.002) | 0.924 (0.853, 1.001) |
| + CO | 1.112 (1.049, 1.178) | 1.066 (1.027, 1.107) | 1.242 (0.954, 1.618) | 1.082 (1.004, 1.166) | - | 0.921 (0.868, 0.977) |
| + O_3_ | 1.109 (1.018, 1.207) | 1.054 (1.006, 1.105) | 1.154 (0.874, 1.523) | 1.014 (0.916, 1.122) | 1.001 (0.999, 1.002) | - |


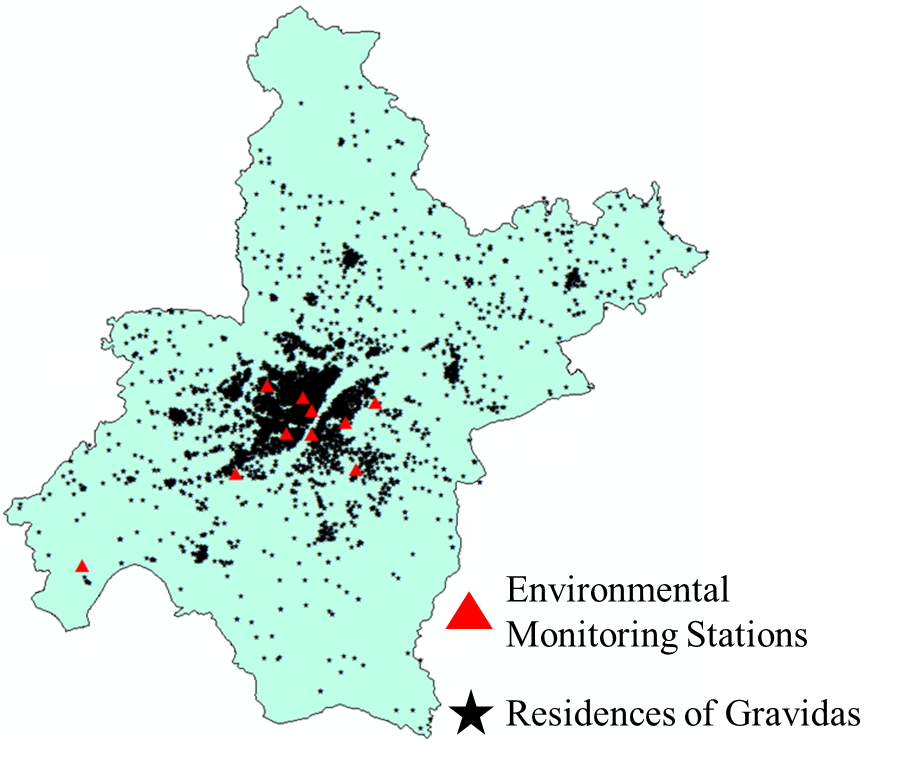


Fig. S1 The locations of environmental monitoring stations and residences of gravidas

1. *** *Corresponding author.* *Department of Obstetrics and Gynecology, Wuhan Children’s Hospital (Wuhan Maternal and Child Healthcare Hospital), Tongji Medical College, Huazhong University of Science and Technology, Wuhan 430014*

   E-mail address:  [zhangyan_wch@163.com](mailto:Zhongyy_WCH@163.com) (Y. Zhang)

   **** *Corresponding author.* *Department of Occupational and Environmental Health, School of Public Health, Wuhan University, Wuhan 430071*

   E-mail address: weizhu@whu.edu.cn (W. Zhu)

   ^1^ Faxue Zhang and Xupeng Zhang are co-first authors who contributed equally to this paper. [↑](#footnote-ref-1)
